# Biochar Stability Revealed by FTIR and Machine Learning

**DOI:** 10.1021/acssusresmgt.5c00104

**Published:** 2025-04-29

**Authors:** Monica A. McCall, Jonathan S. Watson, Jonathan S. W. Tan, Mark A. Sephton

**Affiliations:** † 152935 Earth Science and Engineering, Imperial College London, Exhibition Rd, South Kensington, London SW7 2AZ, United Kingdom; ‡ Grantham Institute for Climate Change and the Environment, 4615 Imperial College London, South Kensington, London SW7 2AZ, United Kingdom; § Viridien Satellite Mapping, Crompton Way, Crawley RH10 9QN, United Kingdom

**Keywords:** infrared spectroscopy, H:C, O:C, molar ratios, modeling, wood, grass, Random Forest

## Abstract

Biochar is a carbon-rich and environmentally recalcitrant material, with strong potential for climate change mitigation. There is a need for rapid and accessible estimations of biochar stability, the resistance to biotic and abiotic degradation in soil. This study builds on previous work by integrating Fourier-transform infrared spectroscopy (FTIR) data with predictive modeling to estimate standard stability indicators: H:C and O:C molar ratios. Lignocellulosic feedstocks were pyrolyzed at highest treatment temperatures (HTT) ranging from 150–700 °C, and all samples achieved H:C < 0.7 and O:C < 0.4 at HTT of 400 °C and above. Several statistical and machine learning models were developed using FTIR spectra. The random forest (RF) models, which incorporated full data preprocessing, yielded the highest accuracy (*R* ^2^ = 0.96 for both ratios) when tested on an unseen feedstock. Variable importance analysis identified spectral regions linked to aromaticity and inversely correlated to C–O stretches in cellulose and lignin as key predictors. The findings of this study verify that FTIR data can serve as a rapid and accurate tool for estimating biochar stability.

## Graphical abstract

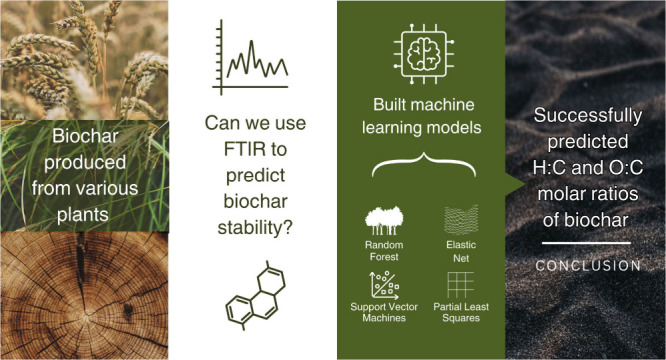

## Introduction

With global surface temperatures now surpassing 1.1 °C above preindustrial levels,^1^ carbon dioxide removal (CDR) methods are gaining significant momentum. Among these, biochar, a carbon-rich and environmentally recalcitrant material produced from the pyrolysis of biomass,^2^ has garnered particular interest, with projected sales exceeding $368 million by 2028.^3^ Biochar can sequester 2.5 GtCO_2_ per year,^3^ while improving soil properties such as nutrient availability, soil organic carbon, crop yields, and water retention.^4^ As its role in climate mitigation and soil amelioration grows, efficient biochar characterization and monitoring techniques must evolve at a pace.

Machine learning, an artificial intelligence application that predicts outcomes without rigorous programming,^5^ is increasingly used across scientific fields,^6^ including biochar research.^7^ By replacing the need for otherwise lengthy and expensive laboratory experiments, machine learning facilitates more time- and cost-effective analyses.^8^ Additionally, accessible coding packages now enable custom-built models, particularly useful for large, complex, and high-dimensional datasets. Several studies have applied machine learning to predict biochar characteristics using known physiochemical properties and pyrolysis conditions,^9^ including predicting heavy metal adsorption,^10^ specific surface area, pore volume,^11^ and elemental composition.^12^ Others have used ^13^C nuclear magnetic resonance spectroscopy (NMR) data alone to estimate the biochar composition.^13^

Fourier-transform infrared spectroscopy (FTIR) is a rapid, non-destructive, and cost-effective method for detecting chemical functional groups in a sample,^14,15^ rendering it ideal for biochar characterization when production details are unknown, such as in artisanal biochar produced in rural areas. FTIR’s ease of data acquisition and information richness render it well-suited for predictive modeling, as its spectra offer abundant data for machine learning.^16^ Despite the advantages of both machine learning and FTIR, their combined applications in biochar research are limited. Wehrle et al.^17^ used support vector machines (SVM) and FTIR to characterize carbon and nitrogen in biochar-based soil amendments. Other studies have paired FTIR with simpler models to predict cation exchange capacity^18^ and other properties of biochar-based fertilizers.^19^ Sajdak et al.^20^ achieved 92–99% accuracy in classifying biomass sources of biochars using FTIR spectra, but there still remains few studies that leverage advanced machine learning techniques for FTIR data.

A notably valuable application of these methods lies in predicting biochar stability or the resistance to biotic and abiotic degradation.^21^ In order to be certified by regulatory bodies, biochar stability is evaluated by its molar ratios of H:C and O:C. Only biochar achieving H:C < 0.7 and O:C < 0.4 is approved for certification.^22,23^ The current standard for determining these ratios is elemental analysis, a process that is lengthy, expensive, and destructive and requires technical expertise. Conversely, FTIR spectroscopy offers a faster and more accessible alternative for producers and certifiers alike. Previous proof-of-concept research demonstrated that simple statistical models could predict H:C and O:C ratios with high accuracy (*R* ^2^ > 0.98) for a single feedstock.^24^ However, to the best of our knowledge, no other studies have explored the use of machine learning to ascertain stability information from FTIR data. This study aims to bridge that gap by applying machine learning techniques to FTIR spectra, enabling rapid and accurate stability predictions.

## Materials and Methods

### Feedstocks

Feedstocks included three species of grass: barley straw (BS), miscanthus grass stems (MG), and rice husk (RH), and three species of wood: chestnut wood (CW), eucalyptus bark (EB), and pine bark (PB). Selected woods included both hard and softwood as well as bark. Lignocellulosic feedstocks were chosen for their popularity in biochar production.^25^ Additionally, one C4 plant (MG) was included. All feedstocks are on the EBC’s list of permissible biomasses for biochar production.^26^ Lastly, these feedstocks represent a range of chemical compositions, summarized in Table 1. Briefly, wood feedstocks are characterized by higher lignin content, whereas greater levels of the biopolymers cellulose and hemicellulose are found in the grasses. Rice husk, known for its silica content, generates a high amount of ash. Extractives, the nonstructural chemical compounds in plants, were in similar ranges across all feedstocks.

**Table 1:** Chemical Composition of Each Raw Feedstock, as Found in the Literature

**Feedstock**	**Type**	**Photosynthetic pathway**	**Cellulose (%)**	**Hemicellulose (%)**	**Lignin (%)**	**Ash (%)**	**Extractives (%)**	**Reference**
Barley Straw (BS)	Grass	C3	34-45	28-36	16-22	4-9	3-8	^27–31^
Rice Husk (RH)	Grass	C3	25-35	18-26	7-31	10-24	5-12	^32–35^
Chestnut Wood (CW)	Hardwood	C3	32-47	18	18-33	0.1-1.2	6-16	^36–38^
Eucalyptus Bark (EB)	Hardwood	C3	23-42	19-23	16-45	5-14	1-10	^39–43^
Pine Bark (PB)	Softwood	C3	17-28	12-23	41-51	1-4.5	4-17	^44–47^
Miscanthus Grass (MG)	Grass	C4	33-50	21-35	17-28	1-8	9	^48–50^

### Biochar Production

All feedstocks were pyrolyzed in a Carbolite Gero tube furnace at 5 °C/min heating and cooling rates under an inert N_2_ atmosphere (1 L/min flow rate). The highest treatment temperature (HTT) ranged from 150–700 °C in 100 °C increments, with samples held at HTT for 30 min. Because significant compositional changes of biochars arise between 250 and 350 °C,^24^ samples were pyrolyzed at these temperatures as well. BS biochar was additionally produced at 150, 450, 550, and 650 °C. Resultant biochars were then weighed and homogenized to ensure sample homogeneity in the FTIR analysis.

### FTIR Specifications

All biochar and feedstocks were analyzed using attenuated total reflectance FTIR (ATR-FTIR) on a Nicolet 5700 Spectrometer. Samples were run in triplicates of 128 scans, in the mid-infrared region of 3700–550 cm^–1^ at 4 cm^–1^ resolution. Spectra were baseline corrected using Spectragryph software using the advanced adaptive baselining method.^51^

### Elemental Analysis

Elemental analysis was conducted by Sercon Analytical Ltd. on a Europa Scientific Elemental Analyzer coupled with Isotope Ratio Mass Spectrometry (EA-IRMS). Twenty percent of samples were duplicated and averaged, and the instrument has a relative standard deviation of 2%.

### Model Development and Evaluation

#### Data Partitioning

Model development and analysis were conducted in R (version 3.4.0) using the caret package.^52^ The predictor variables for the models were the absorbance values at each particular wavenumber in the FTIR spectra. The response variables were H:C and O:C respectively, chosen for their use in the biochar certification process. FTIR data was divided into training (∼80%) and testing (∼20%) sets. The training set included spectra from RH-, CW-, EB-, PB-, and MG-derived biochars, while the test set comprised only BS-derived biochars. The test set contained HTTs absent from the training set but within a similar range, allowing for evaluation of model performance on unseen feedstocks and temperatures.

#### Preprocessing

Data preprocessing, a collection of techniques used to refine data quality, is an integral step for improving prediction accuracy in machine learning.^53^ Normalization, a common practice in FTIR analysis, was performed using a min-max method,^54^ where each spectrum was transformed such that the highest absorbance peak was equivalent to 1. Scaling involved standardizing each predictor variable by dividing it by the standard deviation across all samples. Lastly, principal component analysis (PCA), a dimensionality reduction technique, was applied to address the large number of predictor variables by transforming them into uncorrelated principal components (PCs).^52^ The PCA threshold was set to 95%, ensuring that only the most relevant PCs that cumulatively explain 95% of the variance in the predictors were retained. Models were developed by using different preprocessing combinations to identify optimal techniques.

#### Model Training and Hyperparameter Tuning

To maximize performance while addressing the limited number of observations, K-fold cross-validation was employed in model training.^55^ This involved splitting the training data into K = 10 equal “folds”, where K-1 folds are used to predict the left-out fold as a temporary test set, allowing for the model to be trained and applied to K different datasets.^56^ Hyperparameter tuning was performed for each model and preprocessing combination using a grid search approach. This involved setting up an initial grid space and step size, which was then continuously refined to focus on optimal ranges and increase granularity.^57^ The grid search was completed when perturbations in hyperparameters resulted in negligible improvements in resampling performance (change in root mean square error (ΔRMSE) < 0.01 across all 10 cross-validated resamples). To ensure reproducible results, the set.seed­() function was used to hold the composition of the folds constant throughout model training.^52^

Various model algorithms, both statistical and machine learning, were tested separately for the H:C and O:C predictions. A supervised statistical method, partial-least-squares regression (PLSR), was chosen for its prior success in other FTIR related studies.^18,19^ Elastic net regression, a variable selection and regularization model, is useful when the number of observations is smaller than the number of predictors.^58^ Random Forest (RF) uses a decision tree algorithm which utilizes bootstrap aggregation and randomization of predictors to create many decision trees and achieve highly accurate predictions.^59^ Lastly, support vector machine (SVM) models generalize well to unseen data by maximizing the margin between data points and decision boundaries.^60^ The machine learning algorithms chosen are widely used for regression analysis in other fields, are simple to implement and tune, do not require excessive processing power, and are relatively easy to interpret.

#### Model Evaluation

Model performance was assessed by comparing the cross-validated resampling results using the coefficient of determination (*R* ^2^) and RMSE as evaluation metrics. Following cross-validation, the final models were applied to the unseen test set to predict the H:C and O:C ratios. The variable importance of the best-performing models (as determined by *R* ^2^ and RMSE) was further explored to identify the regions of the FTIR spectra most influential in driving model predictions.

## Results and Discussion

### Elemental Analysis

The van Krevelen diagram in Figure 1 illustrates the relationship between molar H:C and O:C ratios across all feedstocks and biochars. There is a clear trend of reduction in both ratios with increasing temperature. The unpyrolyzed feedstocks have an average H:C and O:C ratio of 1.32 ± 0.15 and 0.68 ± 0.09 respectively, demonstrating large variability in the chemical composition of the starting materials. In contrast, at the HTT of 700 °C the H:C ratio converges to an average of 0.23 ± 0.02, and the O:C ratio converges to an average of 0.09 ± 0.04 across feedstocks. This indicates that while initial composition has a substantial effect on both molar ratios, as HTT increases, the ratios become more dependent on temperature than starting material.^61,62^ Some feedstocks began at relatively lower ratios, reflective of their starting compositions. For example, the unheated lignin-rich PB had an H:C value of 1.17 and an O:C value of 0.53, indicating it had lower ratios than those of cellulose-rich BS pyrolyzed to 250 °C. However, the two have nearly identical H:C ratios at 700 °C, implying that BS underwent a more gradual degradation rate compared to PB. All biochars produced at HTT ≥ 400 °C met the criteria determined by EBC to certify biochar as suitable for soil amendment^22,23^ regardless of starting material. CW biochar produced at 700 °C had the lowest ratios of all the samples (H:C = 0.2, O:C = 0.05), with an estimated carbon storage of 574 g/kg after 100 years in soil, as determined by the IBI carbon storage classification tool.^63^ Lastly, the figure suggests a high correlation between O:C and H:C. However, we caution the use of this relationship and advise it may only be used as an estimation limited to lignocellulosic feedstocks, as the literature shows examples where non-lignocellulosic feedstocks do not display the same linear correlation.^64^

**Figure 1:**
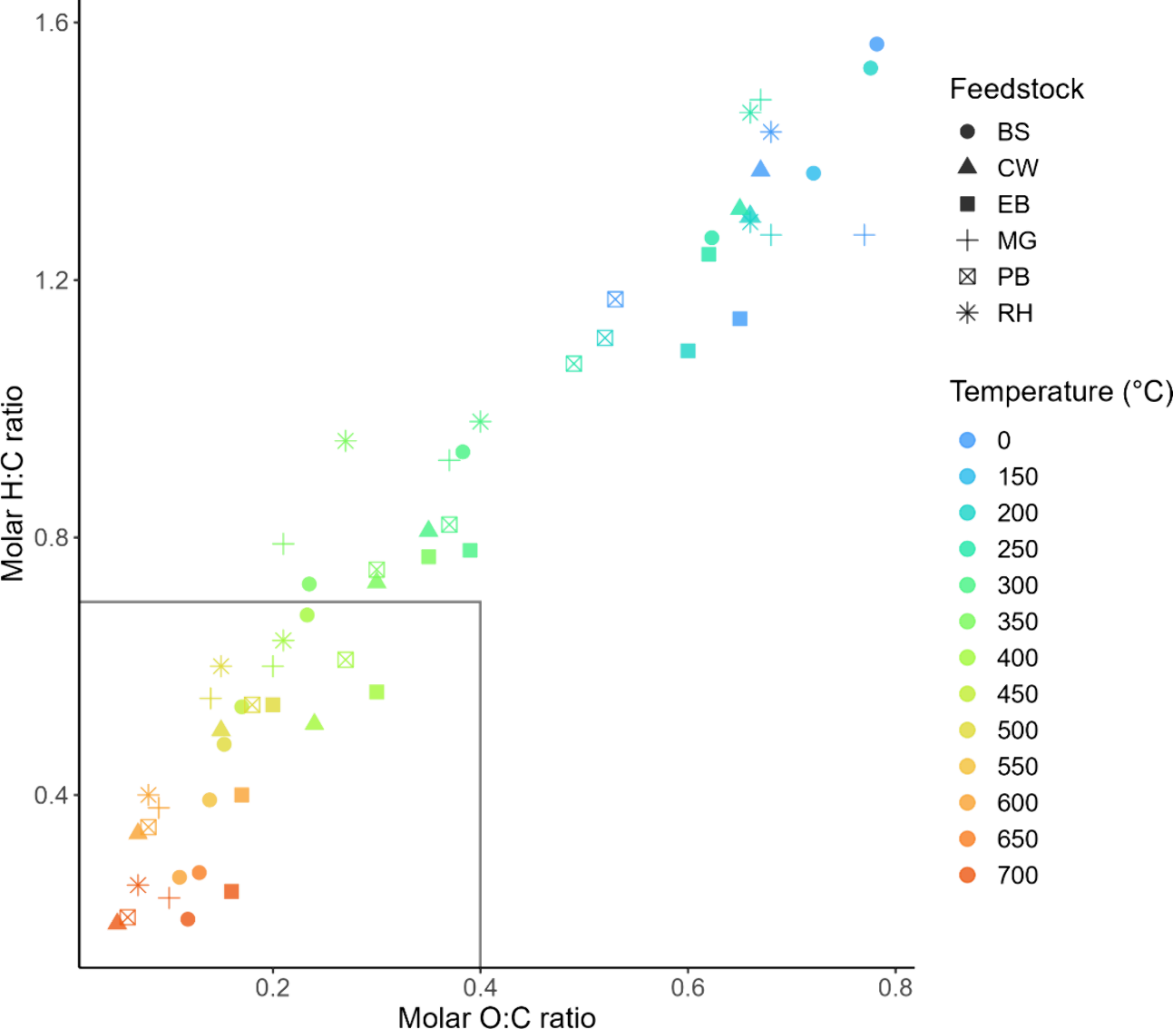
Van Krevelen diagram of both H:C and O:C molar ratios of biomass and biochars produced from the various feedstocks over a range of pyrolysis HTT. The black rectangle represents the ratios determined by EBC and IBI to certify biochar as suitable for soil amendment.^22,23^

### FTIR Spectra

The fingerprint region of the FTIR Spectra of all biochar samples and starting materials can be found in Figure 2. Spectra containing higher wavenumbers can be found in the Supporting Information.

**Figure 2:**
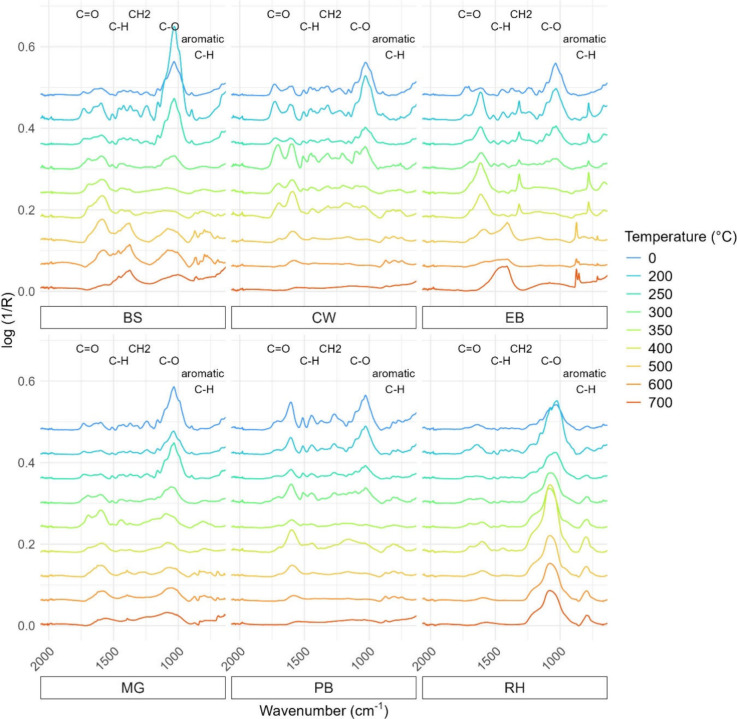
ATR-FTIR spectra of all biochars and starting materials; each spectrum is an average of triplicates for illustrative simplicity and is displayed on a common, offset *y*-axis. HTT is denoted by color, and common peak assignments are noted above their respective peaks.

The common peaks and their assignments are summarized in Table 2. For all feedstocks, peaks around 1700 cm^–1^ are indicative of acetyl, ester, carboxyl groups in hemicellulose and lignin^65–67^ and decrease with temperature. Aromatic skeletal vibrations at ∼1510 cm^–1^ are present at low HTT but are removed around 200–300 °C for the grass-derived biochars and after 400 °C in the woods. The presence of the C–O stretch at 1030 cm^–1^ dominates the spectra of the unheated biomasses and dampens with temperature across all samples except for RH, where this peak remains prominent at 700 °C. As RH is recognized for its high silica content,^35^ this is likely due to a signal of the Si–O–Si stretch at the same wavenumber, corroborated by an additional Si–O–Si bend present at 815–790 cm^–1^.^68^ Peaks corresponding to aromatic C–H bends began to form particularly from 400–600 °C, signaling the formation of stable aromatic rings.^69^ However, peaks in this region diminish in most feedstocks at 700 °C as the high HTT drives off the majority of hydrogen, leaving only graphitic carbon remaining.^70^ The one exception is EB-derived biochar, where the out-of-plane C–H bend peak at 785 cm^–1^ is present from 250–400 °C and degraded above 400 °C. This could be attributed to condensed tannins^71^ or polyphenolic compounds such as the flavonoid quercetin, which has a characteristic peak in this region and is present in eucalyptus.^72^ BS and EB are also the only spectra to retain a C–H bend peak around 1430 cm^–1^ at 700 °C.

**Table 2:** Common Peaks in the ATR-FTIR Spectra of All Biochars with Chemical Assignments^a^

**Peak** **(cm** ^**–1**^ **)**	**Chemical assignment**	**Reference**
**1750–1650**	C=O stretch	^76^
**1595–1512**	aromatic skeletal vibration	^74^,^77^,^78^
**1462–1316**	C–H bend and CH_2_ wag + O–H bend vibrations	^74^,^76–79^
**1247–1030**	C–O stretch in lignin + cellulose	^74^,^76^,^78–80^
**870–680**	aromatic C–H bend	^69^,^81^,^82^
**815–790**	Si–O–Si bend	^68^
**750–650**	C–OH out of plane bend	^79^

a More detailed assignments related to the biopolymers lignin and cellulose have been previously reported.^24^

Differences in hardwoods and softwoods were identifiable. PB, a softwood, contains more G-units and displayed prominent G-unit specific peaks such as the C–O stretch at 1267 cm^–1^ and aromatic C–H bend at 813 cm^–1^.^73^ Meanwhile, both EB and CW, hardwoods, contain a mix of G- and S-units and exhibited more S-unit characteristics peaks, such as the C–O stretch located at 1315 cm^‑1.^ ^74^ Other studies have leveraged this difference and predicted hardwood and softwood contents using FTIR and statistical models.^75^

### Modeling

#### Cross-Validation

To optimize model performance, each model and preprocessing combination were treated as individual models, where hyperparameters were tuned using grid search, a common method for hyperparameter optimization.^83^ The results of hyperparameter tuning and selection for each model are summarized in Table S3 in the Supporting Information. A summary of the *R* ^2^ and RMSE values of cross-validated model training can be found in Figure 3.

**Figure 3:**
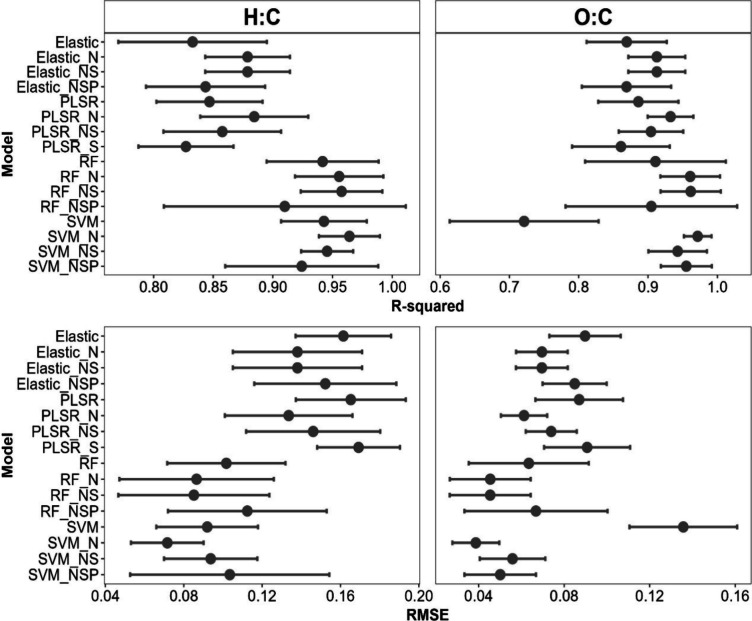
Model comparisons of *R* ^2^ and RMSE for both predicted H:C and O:C ratios on training data. The dot signifies the mean of the cross-validated resamples, and the error bars display the standard deviation. Preprocessing steps are abbreviated to N (normalized), S (scaled), NS (normalized and scaled), and NSP (normalized, scaled, and PCA).

During cross-validation, 50% of the H:C models had a mean *R* ^2^ above 0.9, though preprocessing caused mixed results in model performance. For elastic net and PLSR models, preprocessing improved *R* ^2^ values, aside from scaling in PLSR. For SVM and RF models, normalizing and scaling improved predictions, but adding PCA generated more variability in the cross-validated resamples, as demonstrated by wider error bars. For the H:C model, the SVM_N model performed most optimally (mean *R* ^2^ = 0.96 ± 0.02), followed by the RF_N and RF_NS models (both mean *R* ^2^ = 0.96 ± 0.04). The SVM_N training model also had the lowest mean RMSE of 0.04 ± 0.02.

Sixty-nine percent of O:C models attained mean *R* ^2^ values above 0.9. SVM_N performed best with a mean *R* ^2^ = 0.97 ± 0.02 and RMSE of 0.04 ± 0.01. This model also exhibited the smallest standard deviation across all metrics, signifying consistency in the cross-validated resamples. Like the H:C models, the RF_N and RF_NS models also performed well, both achieving a mean *R* ^2^ = 0.96 ± 0.04. The SVM model without preprocessing performed the poorest (mean *R* ^2^ = 0.72 ± 0.1 and RMSE of 0.13 ± 0.03), potentially due to overfitting to noise or irrelevant features in the spectra.

#### Predictions on Test Data

After tuning and training were complete, the models were then deployed on the test data, consisting of FTIR spectra from BS biochar. Scatter plots of the actual molar ratios against predicted ratios are illustrated in Figure 4.

**Figure 4:**
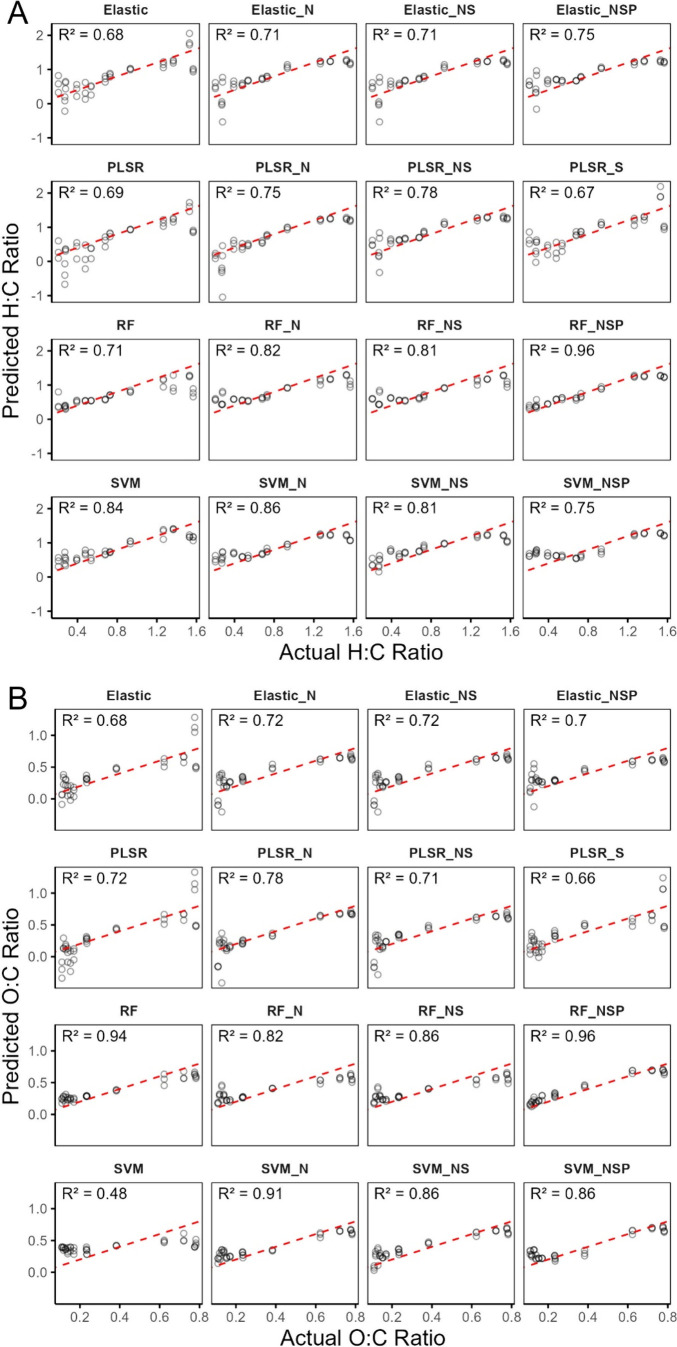
Model predictions of H:C and O:C molar ratios vs actual values on unseen test data, which comprises a new feedstock (BS) and temperature treatments. Preprocessing steps are abbreviated to N (normalized), S (scaled), NS (normalized and scaled), and NSP (normalized, scaled, and PCA).

For the H:C prediction on test data, models that used elastic net algorithms and PLSR algorithms achieved roughly similar results, with mean *R* ^2^ values of 0.71 ± 0.03 and 0.72 ± 0.05 respectively. Models using RF and SVM algorithms produced relatively more accurate results with mean *R* ^2^ = 0.83 ± 0.1 and 0.81 ± 0.05 respectively. The best-performing individual model for H:C was the RF with full preprocessing (RF_NSP), achieving *R* ^2^ = 0.96 and RMSE of 0.15. The same model was also most optimal for the O:C predictions, with *R* ^2^ = 0.96 and RMSE of 0.08. Because the *R* ^2^ value of RF_NSP was the same for H:C and O:C predictions, this signifies that the model was able to equally assess hydrogen and oxygen content information from the FTIR data. The poorest performing algorithm for the O:C predictions was the elastic net, with mean *R* ^2^ = 0.70 ± 0.02, and slightly better was the PLSR, with a mean *R* ^2^ of 0.72 ± 0.05. SVM models performed worse on O:C predictions than H:C, with mean *R* ^2^ = 0 .78 ± 0.19, having the greatest variability due to preprocessing differences across them. Once again, RF models achieved the highest predictive accuracy with a mean *R* ^2^ = 0.89 ± 0.07.

These results indicate that RF is best suited for FTIR data for both H:C and O:C predictions. However, preprocessing can have significant effects, as RF predictions on H:C improved by 35% when implementing the normalization, scaling, and PCA steps compared to the non-preprocessed version. Although improvements on the O:C predictions between unprocessed and RF_NSP results were negligible, it is advised that preprocessing be used on a case-by-case basis. Most models exhibited weaker performance on test data than train data (Figure 3), indicated by lower testing *R* ^2^ (*R* ^2^_test_) compared to the *R* ^2^ of the same model during training (*R* ^2^_train_). This is a common trend, as a meta-review of biochar machine learning studies found *R* ^2^_test_ to be lower than *R* ^2^_train_ in nearly all cases.^9^ This discrepancy is likely due to overfitting, where the model learns the training data too well and fails to generalize to unseen test data.^84^ Figure 4 also reveals that extreme values for molar ratios, particularly H:C values beyond the range of 0.6–1.4 or O:C values outside 0.2–0.7, were more difficult for the models to predict. In FTIR spectra of biochars with low molar ratios (Figure 2), most peaks have been removed or dampened, which is likely why the models have more trouble in this range of ratios.

Though previous work^24^ established that stability data could be accurately predicted from FTIR spectra using one feedstock, the results here indicate that molar ratio predictions can be made with several lignocellulosic feedstocks. There are limited other studies that combine FTIR and modeling to predict biochar characteristics. Lago et al.^18^ attained an *R* ^2^ of 0.81–0.87 for PLSR predictions of biochar cation exchange capacity from FTIR data using 18 feedstocks including non-lignocellulosic materials. De Morais and Silva^19^ also used PLSR and FTIR to assess nutrient information on biochar-based fertilizers and achieved an *R* ^2^ > 0.80 for prediction of nitrogen pools. These relatively lower *R* ^2^ values are consistent with our PLSR results and may indicate that machine learning offers an advantage in place of simpler statistical models, or this could also be attributed to the wider variety of feedstocks in those studies. Zhu et al.^85^ utilized RF models to predict carbon contents of lignocellulosic biochar based on known feedstock chemical composition and pyrolysis conditions and produced an *R* ^2^ of 0.76–0.85. The results of this study indicate that far simpler laboratory analyses in the form of FTIR can be performed to achieve more accurate predictions.

#### PCA Extraction and Variable Importance

RF_NSP was the best-performing model for both H:C and O:C predictions; it was thus the focus of the variable importance analysis. Variable importance helps identify the most influential predictors (i.e., specific wavenumbers) that drive the model’s predictions. However, since PCA was used in preprocessing, PCs replaced individual wavenumbers. Figure 5 presents the PCA results, including the variance explained by each PC, spectral regions with high loadings, and the variable importance rankings for the best-performing model (RF_NSP) in predicting both molar ratios. Notably, the PCA was applied to the training data, which remained consistent for both H:C and O:C predictions.

**Figure 5:**
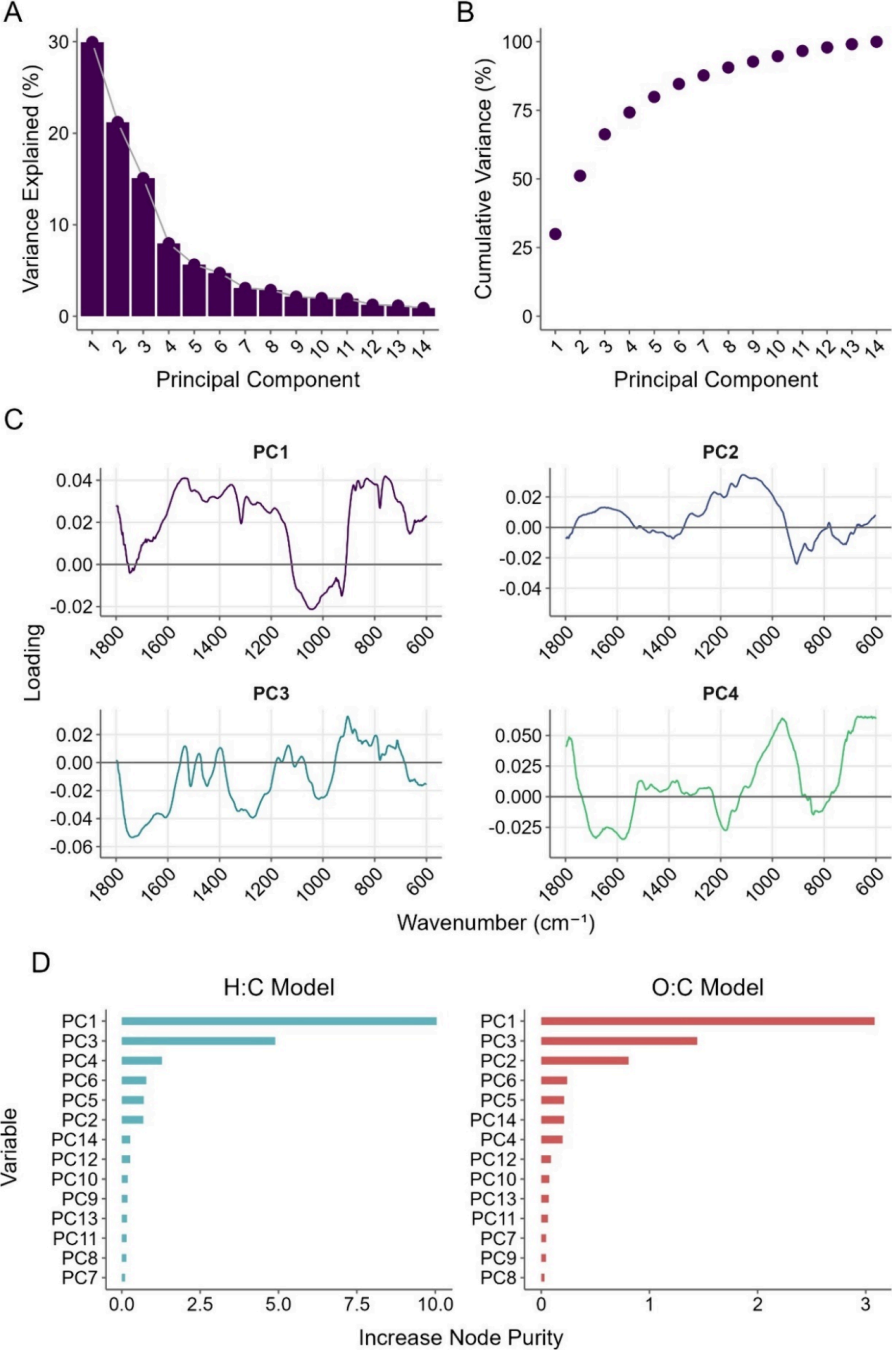
Exploration of PCA used in the preprocessing steps of model training. A) Scree plot showing variance explained of each individual PC, B) Cumulative variance of the principal components, C) Loadings of each wavenumber in the fingerprint region in the first 4 PCs, D) Variable importance of each PC for both the H:C and O:C model predictions as measured by increase in node purity, arranged in order of descending importance.

In the scree plot in Figure 5A, the variance explained by the first four PCs are 30%, 21%, 15%, and 8% respectively, after which subsequent PCs individually contribute <7% of total variance. While the final model retained 14 PCs to reach 95% total variance, Figure 5B confirms that PCs 1-4 accounted for 74% of the cumulative variance. The gradual decay in explained variance suggests that the PCA preprocessing effectively reduced dimensionality for the original complex data. In Figure 5C, the loading values indicate the contribution of each wavenumber to that PC; however, because PCA is an unsupervised method, it is not possible to draw conclusions on the relationship between each PC and molar ratios with loadings information alone. PC1 had its highest positive loadings in the spectral regions of 1530 cm^–1^ (aromatic skeletal vibrations),^77^ 1370 cm^–1^ (C–H symmetric deformation),^76^ and 830–760 cm^–1^ (aromatic C–H bending and wagging).^69^ PC1 therefore found signals associated with aromaticity most important in describing the data variance. PC1 had strong negative loadings in 1060–1000 cm^–1^ (C–O stretch of cellulose and lignin)^79^ and 930 cm^–1^ (C–O–C ring vibration).^86^ Samples with peaks in these regions reflect unpyrolyzed material still rich in lignin and cellulose, making them inversely correlated to the positive aromaticity loadings. PC2 was influenced strongly by positive loadings at 1100 cm^–1^ (asymmetric C–O–C stretch in cellulose)^74^ and a negative loading at 900 cm^–1^ as seen similarly on PC1. PC3 was dominated by negative loadings in 1740–1550 cm^–1^ (carbonyl stretches)^76^ and 1400–1200 cm^–1^ (C–H deformation moieties).^79^ Lastly, the top positive loadings of PC4 included 1800 cm^–1^ (carboxyl groups),^87^ 960 cm^–1^ (out-of-plane aromatic bending),^88^ and 700–600 cm^–1^, although the lower end of the spectrum could be attributable to a rising baseline caused by scattering.

The variable importance was assessed by the increase in node purity metric (IncNodePurity), which quantifies the ability of each predictor variable to separate the data into homogeneous subgroups.^89^ IncNodePurity is a unitless measure reflecting the cumulative reduction in variance achieved by splitting that variable. Figure 5D reveals that for the H:C model, the top 3 important variables were PC1, PC3, and PC4 with IncNodePurity values of 10.04, 4.89, and 1.28, respectively, with other PCs having much lower importance. Therefore, PC1 creates the most meaningful and homogenous subdivisions in the data and is most important for prediction, aligning with PC1 capturing the most critical spectral information. Notably, PC2, which mostly represents the asymmetric stretch of C–O–C in cellulose, does not appear in top variables for predicting H:C ratios, meaning it contains less relevant information for predictions of H:C.^90^ For the O:C model, PC1 again demonstrated the highest IncNodePurity of 3.08, indicating a substantial contribution to reducing variance and improving model accuracy. This was followed by PC3 and PC2, which had IncNodePurity = 1.44 and 0.80 respectively. Interestingly, PC3, while explaining only 8% of the variance in the data, was the second-most-important variable for both models. The lowest-ranked PCs have minimal contributions to predictive accuracy. This analysis demonstrates PCA’s utility in data complexity reduction and the identification of the most predictive variables in the models. Sensitivity analyses linking varying numbers of PCs retained in model training to the interpretability of variable importance can be found in the Supporting Information.

#### Model Application

H:C and O:C molar ratios are industry-standard proxies for biochar stability, used by producers, buyers, and regulatory bodies. Certification bodies like the EBC require laboratory analysis for these ratios, which is a costly and time-consuming process. Purpose-built machine learning models can alleviate some of this burden by providing a real-time verification tool, potentially for on-site analysis. Biochar producers could also train their own FTIR models as a production monitoring measure. In field research, models could be integrated into handheld spectrometers, which provide accurate soil sample analyses,^91^ to delivery instant stability predictions. Additionally, there is growing momentum in low- and middle-income countries producing “artisanal” biochar, often using off-grid kilns in rural areas.^92^ These producers lack precise temperature controls and work with heterogeneous feedstocks, making quality assessment challenging. FTIR readings combined with machine learning could provide rapid stability insights without knowledge of production conditions.

However, as with all machine learning models, generalization remains a challenge, and the model performance may vary across different FTIR instruments. To address this, we recommend either centralized laboratory use or expanding training datasets to include diverse instrumentation. Moreover, this study focused exclusively on lignocellulosic feedstocks, and further research is needed to assess generalization to other materials. Given that FTIR is an information-rich technique, future studies could explore predicting other biochar properties that are directly linked to functional groups in the spectra such as proximate analysis outcomes. As biochar technology continues to evolve, advanced analytical tools such as the methods outlined here are essential for ensuring quality control and driving further innovation in the field.

## Conclusions

This research investigated the prediction of biochar H:C and O:C molar ratios by training various machine learning models on FTIR spectral data. Expanding on previous studies, this work incorporated a diverse range of lignocellulosic feedstocks and evaluated models on an independent dataset comprising a previously unseen feedstock and HTTs. While the majority of models achieved *R* ^2^ > 0.9 on training data, performance declined on test data, highlighting limitations in generalization. RF models, when combined with normalization, scaling, and PCA preprocessing, were the most optimal for predicting biochar stability information from FTIR data (*R* ^2^_test_ = 0.96). Variable importance analysis determined that PC1, which captures spectral variance associated with aromaticity and C–O stretches in cellulose and lignin, was the most significant predictor for H:C and O:C. FTIR is already widely used in biochar research as a rapid and cost-effective qualitative method. This study demonstrates that when integrated with machine learning, FTIR data can be transformed into a powerful predictive tool for biochar stability.

## Supplementary Material





## Notes

FTIR data (XLSX)
Elemental data, FTIR spectra (3700–2500 cm^–1^), estimated biochar carbon storage, hyperparameter tuning, sensitivity analysis, and correlation plots between PC1 and other variables (PDF)
