# Supplementary material for: Biochar Stability Revealed by FTIR and Machine Learning
Source: ACS Sustain Resour Manag. 2025 Apr 29;2(5):842–52. doi: 10.1021/acssusresmgt.5c00104 (PMC12105012; doi:10.1021/acssusresmgt.5c00104)
Supplement: Supplementary file 2 [file rm5c00104_si_002.pdf]

Supporting Information

Title: Biochar Stability Revealed by FTIR and Machine Learning

Authors: Monica A. McCall [a, b*], Jonathan S. Watson [a], Jonathan S.W. Tan [c], and Mark A. Sephton [a]
[a] Earth Science and Engineering, Imperial College London, Exhibition Rd, South Kensington, London, SW7 2AZ United Kingdom
[b] Grantham Institute for Climate Change and the Environment, Imperial College London, South Kensington, London, SW7 2AZ, United Kingdom
[c] Viridien Satellite Mapping, Crompton Way, Crawley, RH10 9QN, United Kingdom

*Corresponding author: monica.mccall@imperial.ac.uk

Contents

Supporting Information........1
- Data........2
- Figure S1........2
- Figure S2........3
- Table S1........4
- Table S3........5
- Figures S3........6
- Figure S4........7
- Figure S5........9
- Figure S6........10
- Figure S7........11

Data

All FTIR spectral data used in this study can be found in the file FTIR_data.csv.

Figure S1

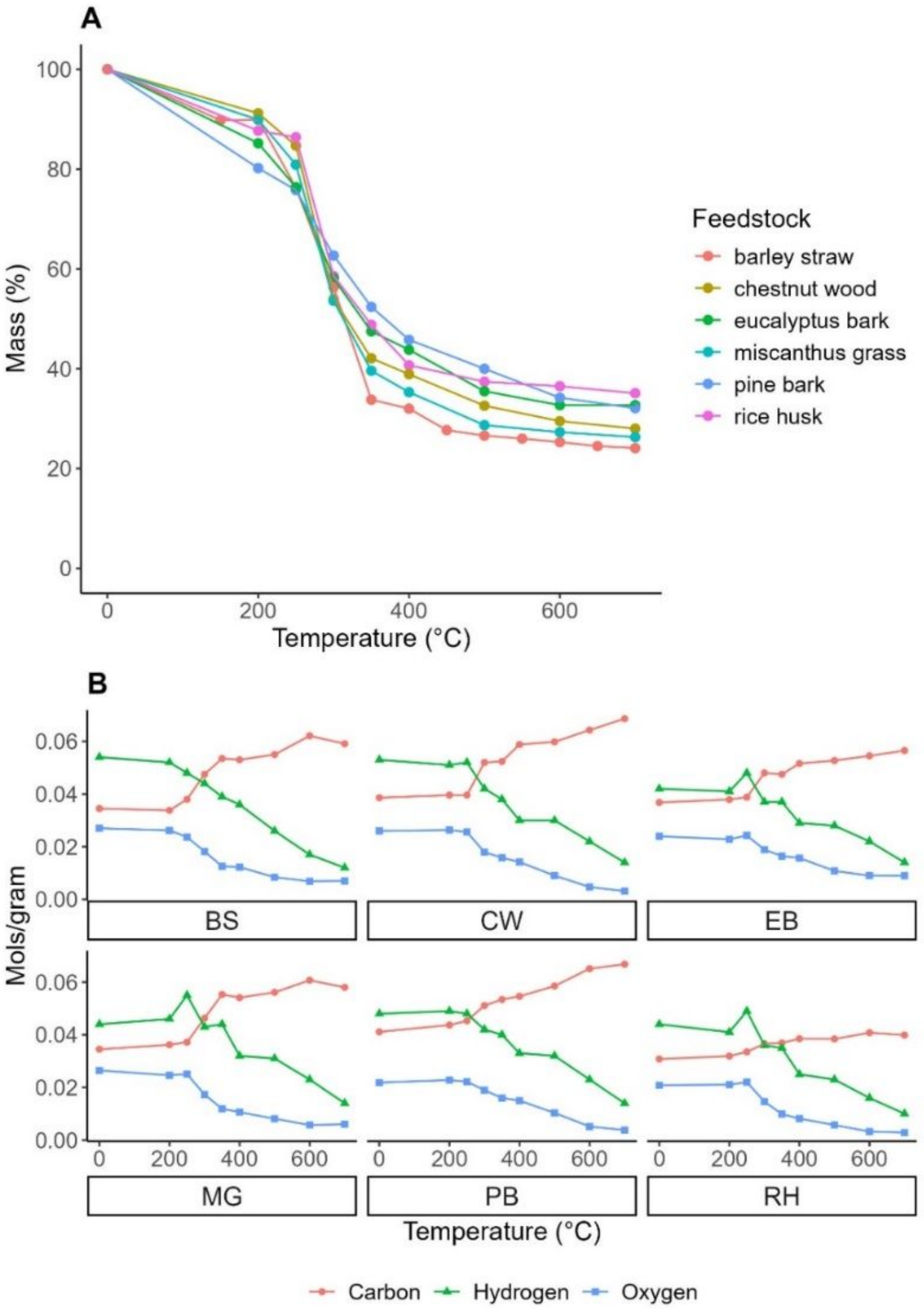

Figure S1: A) Mass loss of biochars derived from various feedstocks at HTT of 150 to 700°C. B) Elemental content of all biochar samples as determined by Elementary Analyzer. Note barley straw biochar data has previously appeared in McCall et al., 2024.

Figure S2

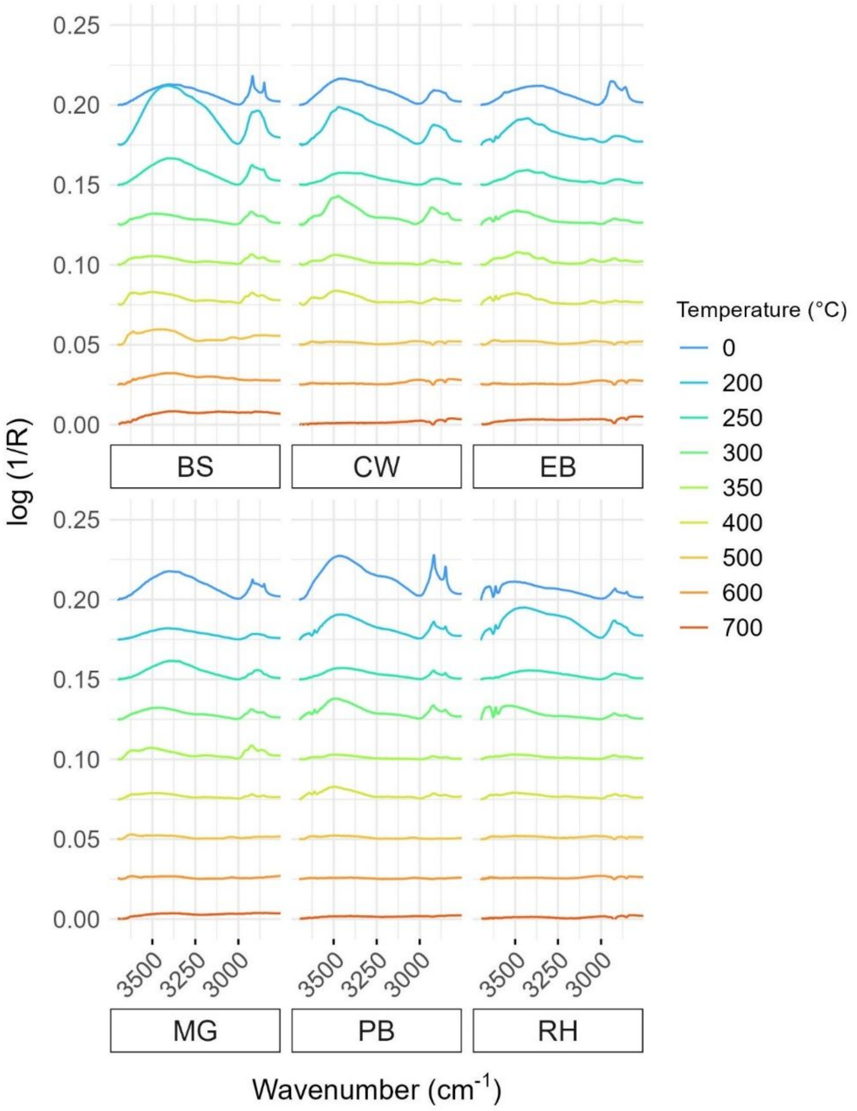

Figure S2: ATR- FTIR spectra of all biochars and starting materials in the region of 3700-2500 cm^{-1}. Each spectrum is an average of triplicates for illustrative simplicity. HTT is denoted by color.

Table S1

Table S1: Carbon storage in g/kg of each biochar produced after 100 years (BC_{+100}). BC_{+100} is used to estimate the long-term soil carbon sequestration potential of a specific biochar. Carbon storage values were determined using the International Biochar Initiative (IBI) classification tool*. Note that biochar samples that scored no carbon storage are excluded from the table.

Feedstock	Temperature (°C)	Carbon (%)	H:C molar ratio	Carbon storage (g/kg) *
**BS**	400	64	0.68	320
**BS**	500	66	0.48	330
**BS**	600	75	0.27	525
**BS**	700	71	0.21	497
**CW**	400	71	0.51	355
**CW**	500	72	0.5	360
**CW**	600	77	0.34	539
**CW**	700	82	0.2	574
**EB**	400	62	0.56	310
**EB**	500	63	0.54	315
**EB**	600	65	0.4	455
**EB**	700	68	0.25	476
**MG**	400	65	0.6	325
**MG**	500	67	0.55	335
**MG**	600	73	0.38	511
**MG**	700	70	0.24	490
**PB**	400	66	0.61	330
**PB**	500	70	0.54	350
**PB**	600	78	0.35	546
**PB**	700	80	0.21	560
**RH**	400	46	0.64	230
**RH**	500	46	0.6	230
**RH**	600	49	0.4	343
**RH**	700	48	0.26	336

* The IBI classification tool estimates BC_{+100} using the H:C value and carbon concentrations (C_{org}) of biochar samples. The tool utilizes extrapolations from decay models developed from medium-term (3 to 5 years) incubation experiments. Further details on the modelling of BC_{+100} can be found in the IBI Carbon Stability Test Method document. The IBI Classification Tool is available at: https://biochar-international.org/resources/biochar-classification-tool/

Reference:

Biochar Carbon Stability Test Method: An assessment of methods to determine biochar carbon stability. International Biochar Initiative. September 20, 2013. Available at https://biochar-international.org/wp-content/uploads/2018/04/IBI_Report_Biochar_Stability_Test_Method_Final.pdf

Table S3

Table S3: Hyperparameter initial search ranges and best tune used in the final version of each model developed

ML Method	Preprocessing	Initial Grid Ranges	Best Tune Value	
			H:C models	O:C models
**Elastic**	None	α=0 to 1<br>λ= 10^{-4} to 10	α = 0.028, λ = 0.018	α = 0.021, λ = 3.0 $\times 10^{-3}$
	Normalization		α = 0.025, λ = 0.1	α = 0.117, λ = 0.01
	Normalization, Scaling		α = 0.025, λ = 0.1	α = 0.122, λ = 5.0 $\times 10^{-3}$
	Normalization, Scaling, PCA		α = 0.05<br>λ = 0.005	α = 0.094<br>λ = 0.005
**PLSR**	None	Components= 0 to 20	ncomp = 12	ncomp = 10
	Normalization		ncomp = 12	ncomp = 13
	Normalization, Scaling		ncomp = 10	ncomp = 8
	Scaling		ncomp =6	ncomp = 8
**SVM**	None	C = 1 to 100<br>σ = 10^{-5} to 10^{-1}	C = 31<br>σ = 2.5×10^{-4}	C = 1.54<br>σ = 4 $\times 10^{-3}$
	Normalization		C = 110<br>σ = 1.9 $\times 10^{-4}$	C = 50<br>σ = 1.3 $\times 10^{-4}$
	Normalization, Scaling		C = 110<br>σ = 5.0 $\times 10^{-5}$	C = 82.5<br>σ = 1.35 $\times 10^{-5}$
	Normalization, Scaling, PCA		C = 12<br>σ = 3.2 $\times 10^{-2}$	C = 28.33<br>σ = 0.02
**RF**	None	mtry* = 40, 536, 804, 1126<br>(p/3, p/2, p x 0.7)	mtry = 750	mtry = 600
	Normalization		mtry = 35	mtry = 30
	Normalization, Scaling		mtry = 35	mtry = 20
	Normalization, Scaling, PCA		mtry = 545	mtry = 950

*In the Caret package, other hyperparameters associated with random forest algorithms such as ntree(# of trees) and nodesize (min size of terminal nodes) are not tuned. P denotes number of predictors (n =1,072)

Figures S3

A

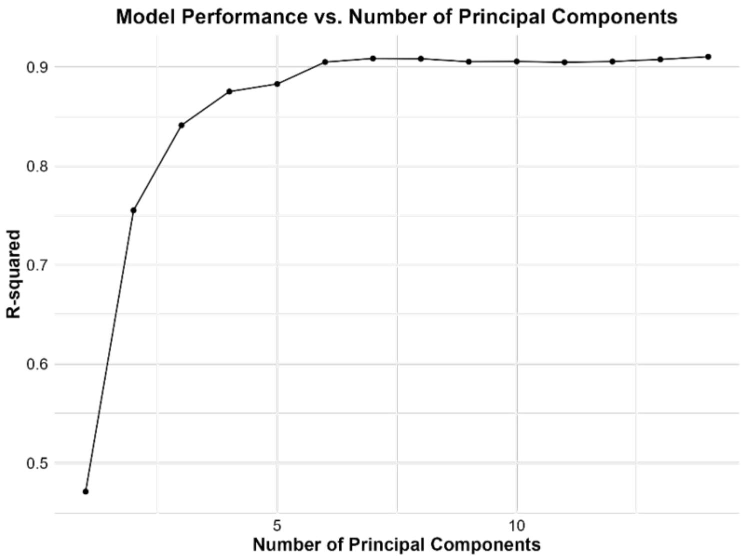

B

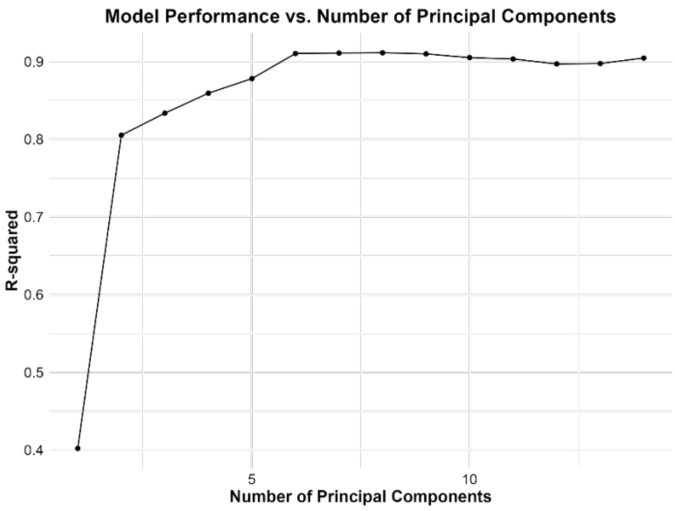

FigureS3: Effect of number of PCs included in the RF_NSP model on model performance as measured by R^2 values. A) Prediction of H:C ratios and B) Prediction of O:C ratios

Figure S4

A

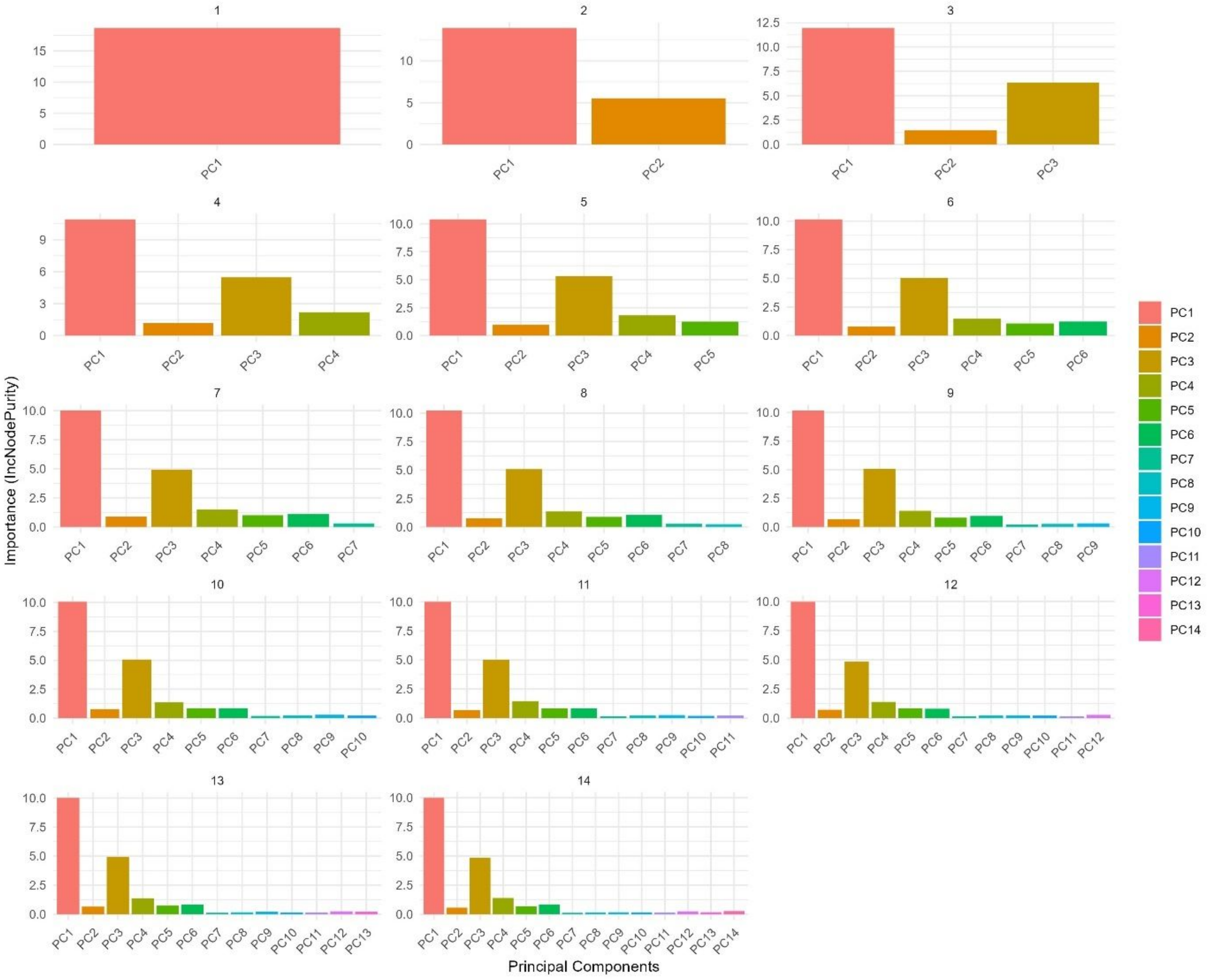

Importance (IncNodePurity)
Principal Components
PC1
PC2
PC3
PC4
PC5
PC6
PC7
PC8
PC9
PC10
PC11
PC12
PC13
PC14

B

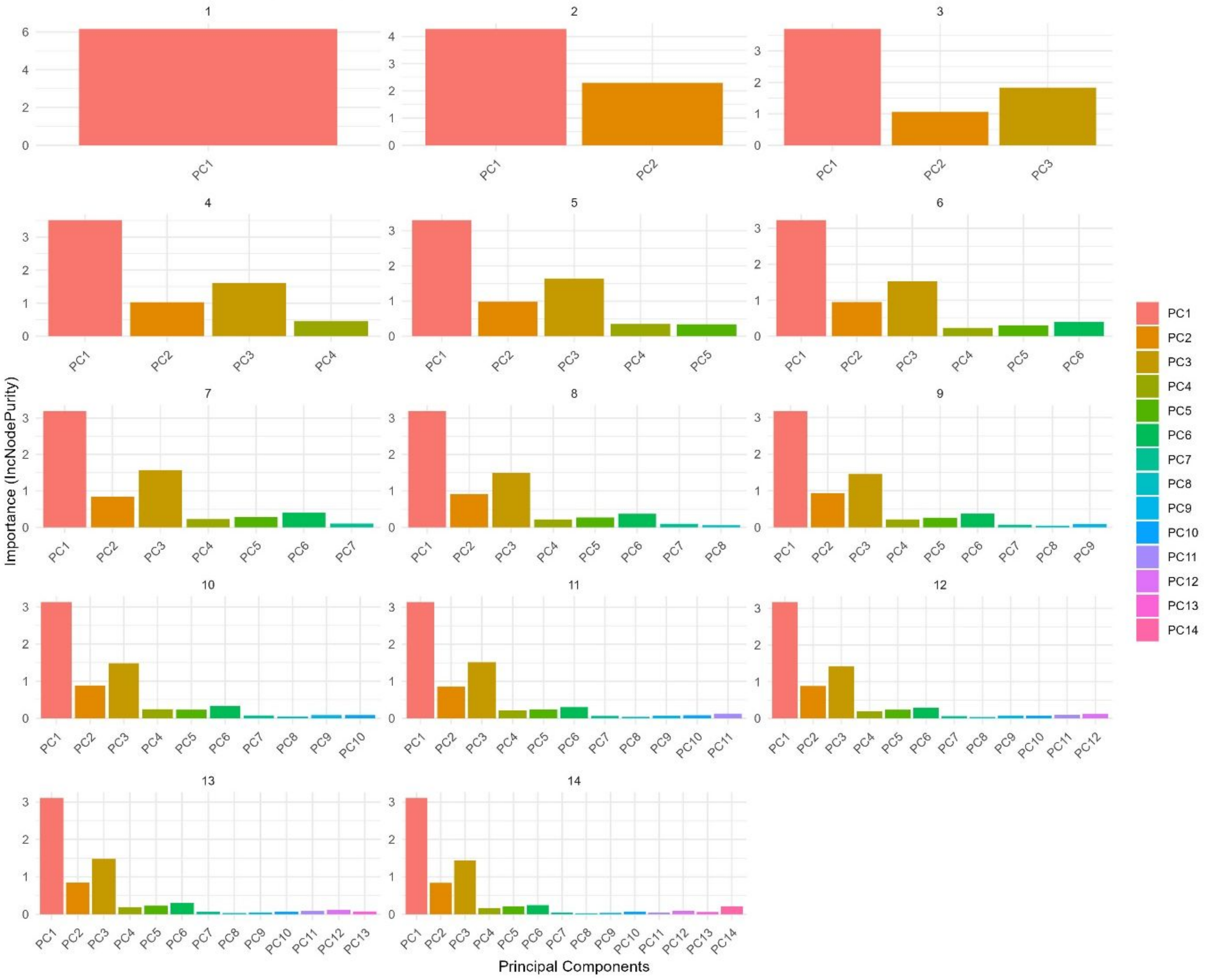

Figure S4: Impact of number of PCs on variable importance in the RF_NSP model used to predict A) H:C ratios and B) O:C ratios. Variable importance of each successive PC added in model training is determined by IncNodePurity, which represents how much each PC contributes to reducing the impurity of the nodes in the decision trees of the random forest, i.e. how well the trees split the data. The higher the IncNodePurity for a component, the more important that component is in making predictions.

Figure S5

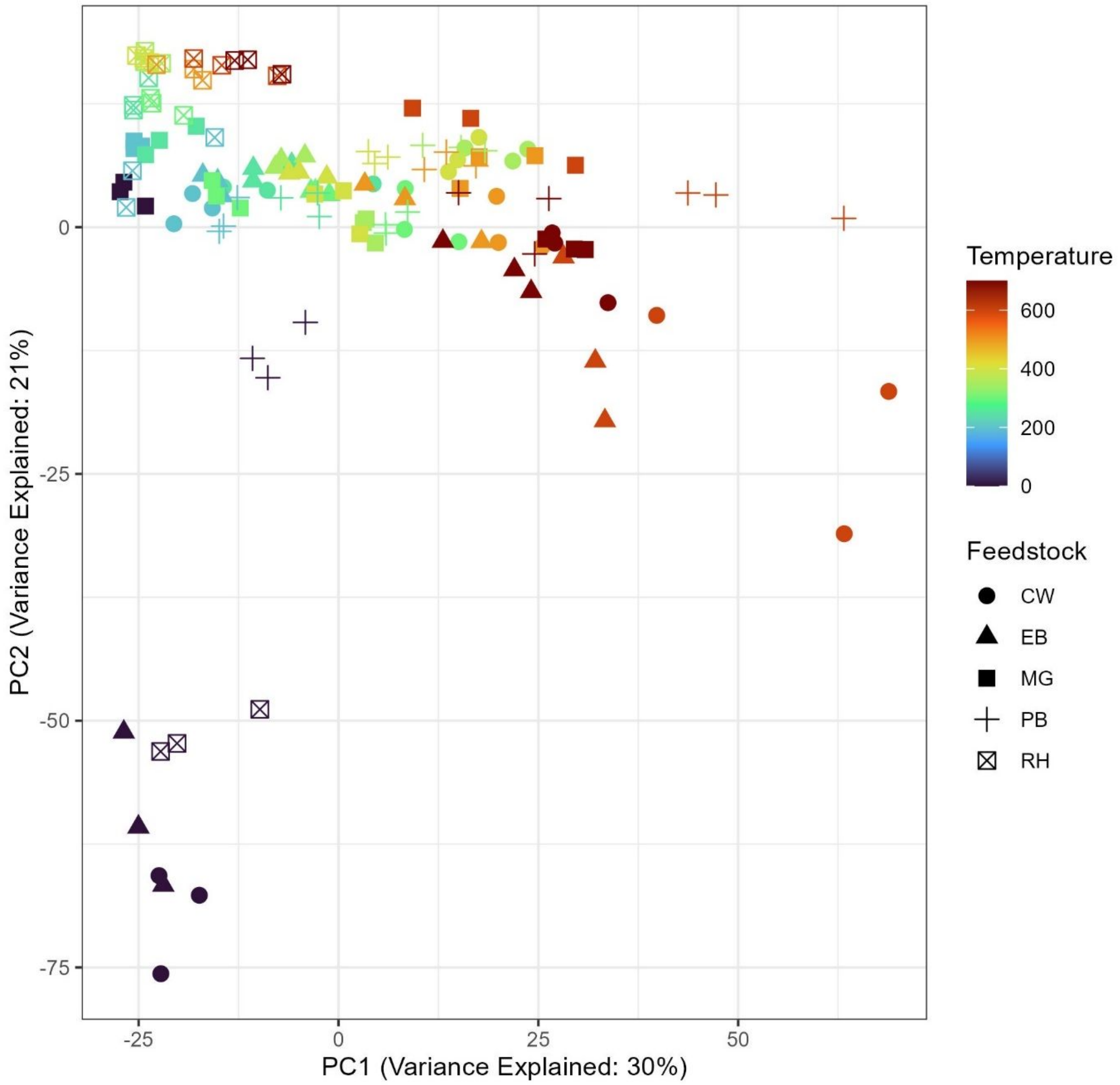

Figure S5: PC1 vs PC2 used in preprocessing of the RF_NSP model in training.

Figure S6

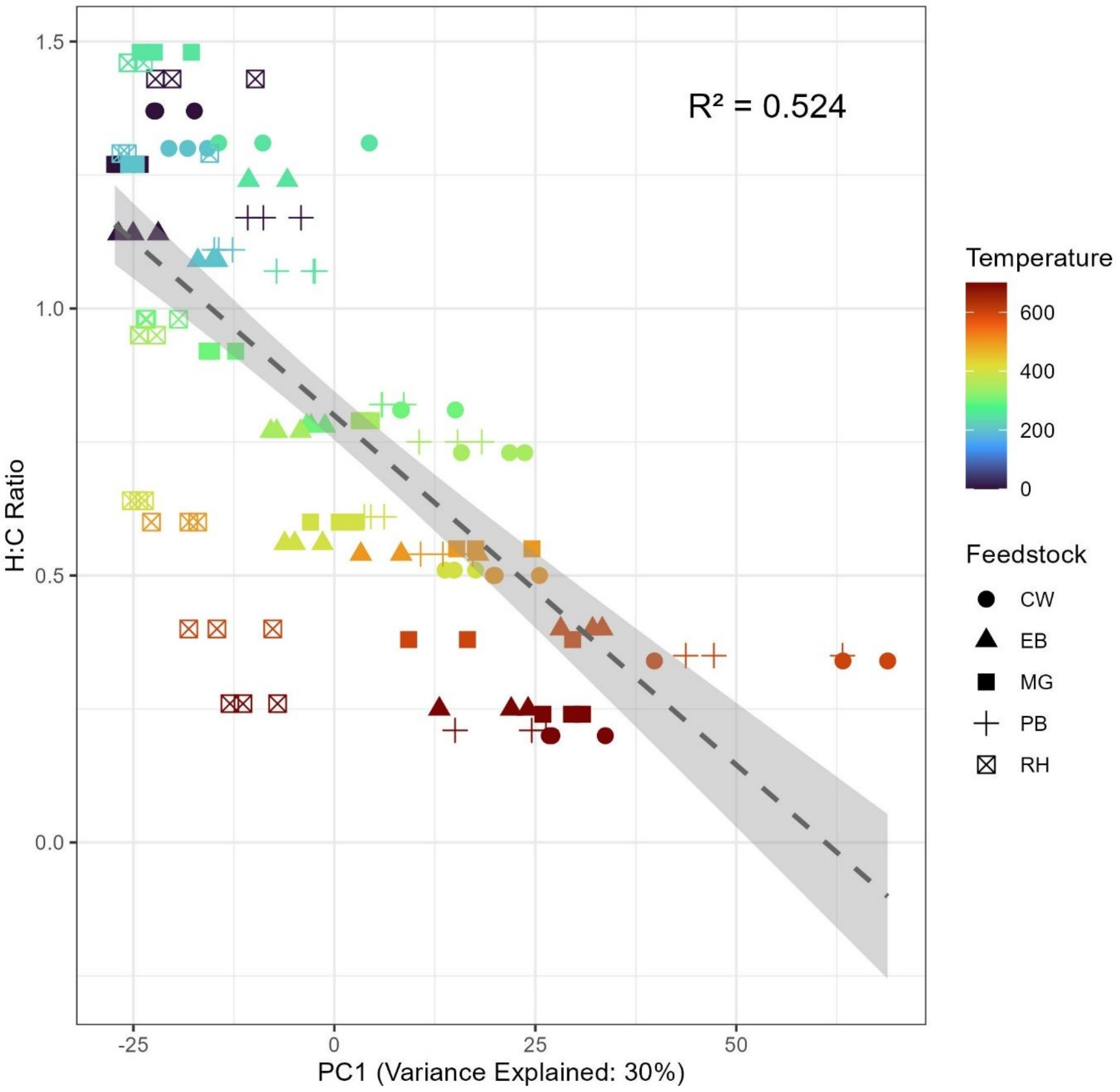

Figure S6: PC1 used in preprocessing of the RF_NSP model in training vs the H:C ratio of each biochar.

Figure S7

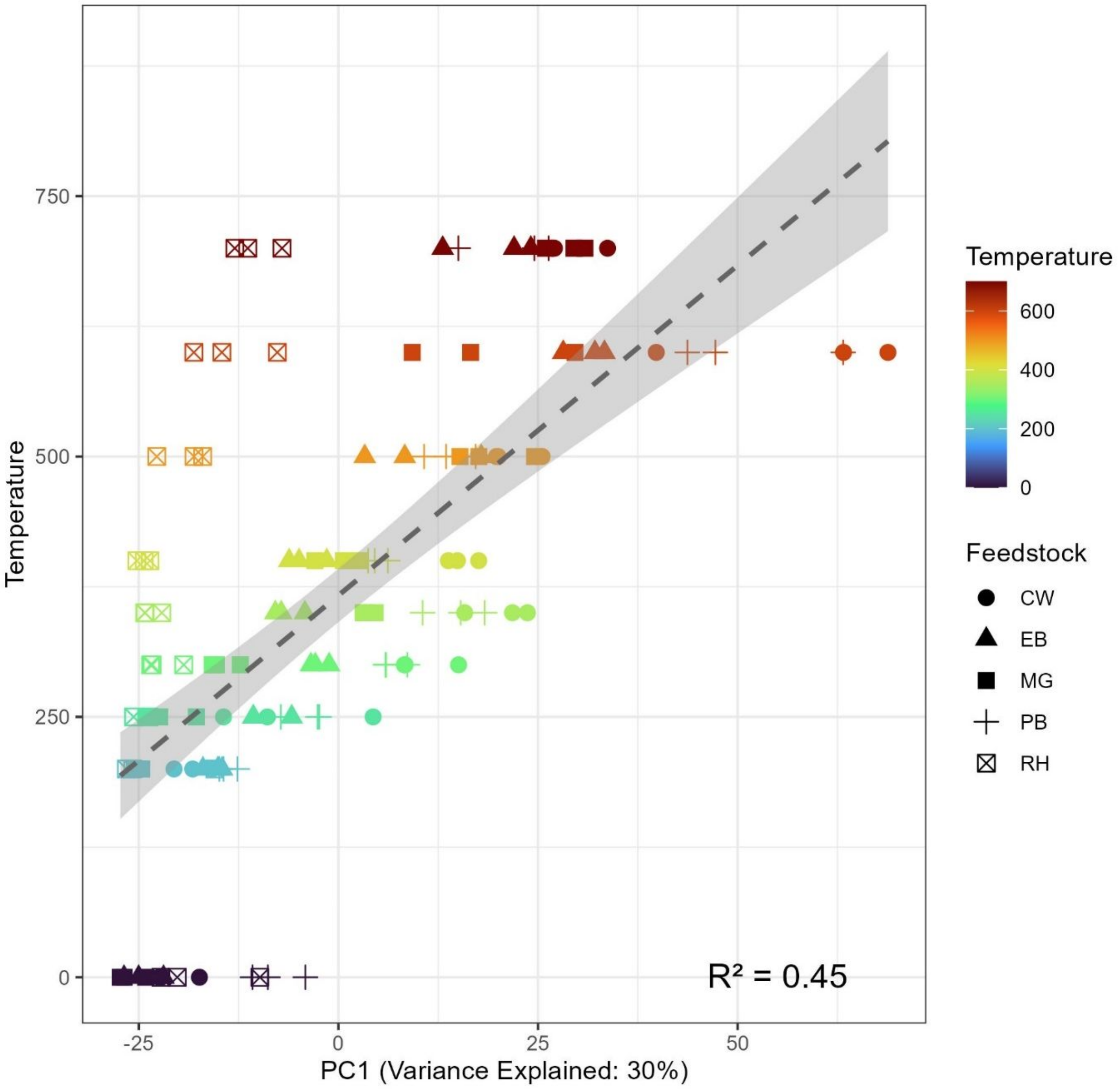

Figure S7: PC1 used in preprocessing of the RF_NSP model in training vs the pyrolysis temperature of each biochar.
